# Predictors of E-Cigarette Use Susceptibility—A Study of Young People from a Socio-Economically Disadvantaged Rural Area in Poland

**DOI:** 10.3390/ijerph16203935

**Published:** 2019-10-16

**Authors:** Dorota Kaleta, Mirosław Niedzin, Agnieszka Jankowska, Kinga Polańska

**Affiliations:** 1 Department of Hygiene and Epidemiology, Medical University of Lodz, 90-647 Lodz, Poland; dkaleta@op.pl (D.K.); mniedzin@wp.pl (M.N.); 2 Department of Environmental Epidemiology, Nofer Institute of Occupational Medicine, 91-348 Lodz, Poland; agnieszka.jankowska@imp.lodz.pl

**Keywords:** e-cigarettes, susceptibility, predictors, youth, socially disadvantaged rural area

## Abstract

Identification of the youth at risk of e-cigarette use is crucial for development of effective prevention strategies. The current study aims at evaluation of predictors of susceptibility to e-cigarette initiation and experimentation among adolescents. This cross-sectional study included 1693 students (non-current users of e-cigarettes) attending 21 schools in Piotrkowski district (a socially disadvantaged rural area in central Poland). The regression models were applied so as to study factors linked to susceptibility to e-cigarette use among never users (*n* = 1054) and ever users (*n* = 639) of e-cigarettes, with susceptibility defined as the absence of a firm decision not to use these products. A high proportion of the youth was susceptible to e-cigarette use (68% of never and 78% of ever e-cigarette users). The adjusted model confirmed the following risk factors: smoking parents and friends (never users: OR = 3.0; *p* < 0.001; OR = 2.0; *p* < 0.05; ever users: OR = 2.2; OR = 2.2; *p* < 0.01), alcohol consumption (never users: moderate drinking OR = 2.9; *p* < 0.001; binge drinking OR = 2.2; *p* < 0.01; ever users: moderate drinking OR = 4.2; *p* < 0.001), cigarette smoking (never users: OR = 14.1; ever users: OR = 11.3; *p* < 0.001), and perception that e-cigarettes are less harmful than traditional cigarettes (never users: OR = 1.8; *p* < 0.001). The youth whose mothers had a medium and high educational level (never users: OR = 2.7; *p* < 0.01; OR = 2.7; *p* < 0.05; ever users: OR = 5.4; OR = 4.4; *p* < 0.001), those who perceived girls who use e-cigarettes as more attractive (never users: OR = 4.1; *p* < 0.001; ever users: OR = 2.9; *p* < 0.01), and secondary school students (ever users: OR = 5.6; *p* < 0.001) had higher odds of susceptibility to e-cigarette use. The youth who had more money per month were less susceptible to e-cigarette experimentation (OR = 0.4; *p* < 0.001). A multi-level intervention approach, considering vulnerable populations, is required to prevent the youth from e-cigarette initiation and experimentation.

## 1. Introduction

Existing epidemiological studies suggest that since electronic cigarettes (e-cigarettes) became available on the market, they have gained unexpectedly large popularity, especially among young people [1,2,3,4]. Attractiveness of these products among the youth is apparent from several factors, including broad advertising and promotion, their use among celebrities, as well as the attractiveness of the product itself [5,6]. Secondly, young people are more prone to dangerous behaviors than adults, and are less aware of health risks or attach little importance to them (because the consequences may appear much later in the future) [7,8,9,10,11]. In addition, the use of e-cigarettes can emphasize attractiveness and independence of young people and, thus, reduce the risk of criticism, as in the case of traditional cigarettes [12,13,14,15,16]. Finally, the lack of legislation or not fully developed and enforced regulations allow easy availability of these products [17,18,19].

Although health consequences of e-cigarette use are not fully confirmed, it is not under discussion that nicotine (present in most e-cigarettes) is a highly addictive substance, one that is especially dangerous for young people [4]. Furthermore, the existing literature reports that e-cigarette use can lead to the initiation of traditional cigarette smoking and result in dual using. The analysis performed by Goniewicz et al. (2018) indicated that exclusive use of e-cigarettes results in measurable exposure to known tobacco-related toxicants [20]. In dual users the toxicant exposure was the greatest and the frequency of combustible cigarette use was positively correlated with tobacco toxicant concentration.

Given the high popularity of e-cigarettes, and because of their health and social consequences, it is crucial to identify the susceptible youth so as to be able to prevent them from initiation or experimentation with these products [4]. Susceptibility to e-cigarette use is defined as the absence of a firm decision not to use them [21] and it is recognized as a predictor of e-cigarette initiation [22]. The analysis performed by Bold et al. (2017) indicated that the e-cigarette susceptible youth were five times more likely to be current e-cigarette users in the following 6 months compared to the non-susceptible youth [22].

Existing studies have pointed out four groups of factors that influence susceptibility to e-cigarette use: risk perception (knowledge about health effects, awareness of addictive effects of nicotine, harmfulness of e-cigarettes compared to traditional cigarettes), social norms and environmental impact (legislation, social acceptance, impact of family and friends), psychological factors (emotional problems, depression), and those related to lifestyle and behavior (negative health behaviors, mainly alcohol consumption and smoking traditional cigarettes) [23,24,25,26,27].

E-cigarettes have been available on the Polish market since early 2008 [28]. The first survey on the use of e-cigarettes in Poland among 20,240 pupils aged 15–24 was carried out in 2010–2011 [28]. The analysis indicated that 24% of the respondents aged 15–19 had tried e-cigarettes and 8% of them had used the product in the last 30 days. Subsequent analyses carried out in 2013–2014 showed a significant increase in the percentage of adolescents aged 15–19 currently using e-cigarettes (to 30% *p* < 0.05) [29]. A study, carried out from November 2014 to May 2015 (before amendment of the law regulating e-cigarette use) covering 3552 students aged 13–19 attending secondary and high schools in Piotrkowski district, showed that 22% of the students experimented with e-cigarettes (they have used them before but not currently), 27% declared current e-cigarette use, and 58% of smokers of traditional cigarettes simultaneously used e-cigarettes [13].

It needs to be highlighted that the predictors of e-cigarette use susceptibility can be country- or even region-specific depending on a variety of factors, including characteristics of the population, social norms, legislation, and preventive measures. The aim of the current study was to investigate various socio-demographic factors, substance use, as well as perception of e-cigarettes that can affect youth susceptibility to e-cigarette initiation and experimentation.

## 2. Material and Methods

### 2.1. Study Design and Population

This cross-sectional study was conducted among the youth (13–19 years) attending all secondary (7–9 years of education) and high (10–12 years of education) schools from Piotrkowski district, which is a socially disadvantaged rural area in Lodzkie voivodship (central Poland). A detailed description of the district and the population has been published previously [13,30,31]. Briefly, in 2017 more than 90% of the residents of the district were people who lived in a rural area (villages with fewer than ten thousands of residents). Furthermore, the unemployment rate for this district was much higher than the national unemployment rate. It also has to be emphasized that the rate of unemployment, in the context of the rural nature of the district, is underestimated as a social problem. Piotrkowski district was also recognized as one with the lowest indicators of social development (including health index, education index, welfare index) compared to other rural districts in Poland.

Data collection was performed between December 2017 and February 2018 (1 year after amendment of the law restricting e-cigarette use) [32]. Of 1979 students from 16 secondary schools and 915 students from 5 high schools that were invited to participate in the study, 2509 agreed to fill in an anonymous questionnaire (participation rate: secondary school—87%, high school—86%). Of this group, 22 (0.9%) respondents were excluded due to missing data on the e-cigarette status (6 participants) and e-cigarette susceptibility (16 participants). In accordance with the aim of the study, the current analysis did not cover 794 current e-cigarette users (the youth who had smoked at least once in the past month); thus, the final sample consisted of 1693 students.

The study was approved by the Bioethics Committee of Medical University in Lodz (RNN/350/17/KE), Education Management Center in Piotrkowski district and directors of the educational institutions. Informed consent was obtained from the study participants or their parents/legal guardians.

### 2.2. Study Variables

A modified version of the questionnaire from the Global Youth Tobacco Survey (GYTS) that consisted of 116 questions was filled in by the students during their regular classes [13].

The current analysis covered two groups of students: never e-cigarette users (to verify susceptibility to smoking initiation) and ever e-cigarette users (to verify susceptibility to smoking experimentation). The never e-cigarette users were the respondents who answered “no” to a question “Have you ever (even once) used e-cigarettes?” Those who answered “yes” to that question but declared not using e-cigarettes in the past 30 days were classified as ever users.

Susceptibility to e-cigarette use was coded on the basis of the answers to the following standardized questions adapted from Pierce et al.: (1) “If one of your friends offered you a cigarette, would you try it?” (2) “At any time during the next 12 months, do you think you will use a cigarette?” [21,22,23,24,33,34]. The students were categorized as non-susceptible if they answered “definitely not” to both questions. Other combinations of the answers (“probably not”, “probably yes”, “definitely yes”) were considered as indicating susceptibility to the use of e-cigarettes.

The evaluated predictors of e-cigarette use susceptibility were similar to those assessed in our previous analysis, which focused on traditional cigarettes [30]. Briefly, we considered socio-demographic variables (youth characteristics: sex, age, school grade, money available per month; and parental characteristics: educational level of the parents) and variables related to smoking of traditional cigarettes and the use of e-cigarettes (smoking status, parental and friends’ smoking and e-cigarette use, perception of attractiveness and harmfulness of the products and information about the ban on smoking at home and at school). Alcohol consumption by the students was also evaluated.

### 2.3. Statistical Analysis

The analyses were conducted using STATISTICA Windows XP version 12.0 (StatSoft Poland Inc., Tulsa, OK, USA). First, the descriptive statistics and distribution of the study variables were presented. To identify predictors of e-cigarette susceptibility the univariate and multivariate logistic regression analyses (with the results presented as odds ratio (OR) and 95% confidence intervals (95% CI)) were performed. The variables with p-values of 0.1 or less from the univariate analysis were included into the multivariate model. Child age as a factor strongly correlated with school grade was not included in the final analysis. The final model was checked for fitness using the Hosmer–Lemeshow goodness of fit test. A p-value below 0.05 was considered statistically significant.

## 3. Results

### 3.1. Characteristics of the Study Population

The current analysis covered slightly more boys than girls (55% vs. 45%) (Table S1). Most of the study participants were attending secondary schools (70%). A high proportion of the youth indicated ever (32%) and current (27%) smoking. Similarly, a very high percentage declared that they had friends who smoked traditional cigarettes (78%) and used e-cigarettes (93%). Only 30% of the respondents were non-alcohol drinkers. Almost half of the study population shared an opinion that e-cigarettes were as harmful as traditional cigarettes.

### 3.2. Susceptibility to E-Cigarette Use

A very high proportion of the youth were susceptible to e-cigarette use (68% of the never e-cigarette users and 78% of the ever e-cigarette users), with higher percentages observed among younger students, those who had better educated parents, those who smoked traditional cigarettes, and those who declared alcohol consumption (Table 1 and Table 2). A higher proportion of the susceptible youth was also observed among those who indicated having parents and friends who smoked traditional cigarettes.

Results of the univariate and multivariate logistic regression analyses of the predictors of e-cigarette use susceptibility are presented in Table 1 and Table 2 and in the Supplementary Materials (Table S1—a combined analysis for the never and ever e-cigarette users). The adjusted model confirmed the following risk factors for e-cigarette initiation (among the never e-cigarette users): smoking parents (OR = 3.0; *p* < 0.001) and friends (OR = 2.0; *p* < 0.05), moderate (OR = 2.9; *p* < 0.001) and binge drinking (OR = 2.2; *p* < 0.01), current smoking of traditional cigarettes (OR = 14.1; *p* < 0.001), and perception that e-cigarettes are less harmful than traditional cigarettes (OR = 1.8; *p* < 0.001). The youth whose mothers had a higher educational level (OR = 2.7; *p* < 0.01 for high school and OR = 2.7; *p <* 0.05 for university) and those who perceived females using e-cigarettes as more attractive had higher odds of susceptibility to e-cigarette use (OR = 4.1; *p* < 0.001). 

The profile of susceptibility to e-cigarette use among the ever users was similar to that observed among the never users (smoking parents: OR = 2.2; *p* < 0.01 and friends: OR = 2.2; *p* < 0.01, current: OR = 11.3; *p* < 0.001 and former: OR = 2.7; *p* < 0.001 cigarette smoking, moderate alcohol drinking: OR = 4.2; *p* < 0.001, medium and high maternal level of education: OR = 5.4; *p* < 0.001 and OR = 4.4; *p* < 0.001, respectively, and opinion that girls who use e-cigarettes are more attractive: OR = 2.9; *p* < 0.01) (Table 2). The secondary school students were more susceptible (OR = 5.6; *p* < 0.001) and those who had more money per month were less susceptible to e-cigarette experimentation (OR = 0.4; *p* < 0.001).

## 4. Discussion

The current study is one of the first to have investigated a comprehensive set of factors that affect susceptibility to e-cigarette use among the youth from a socio-economically disadvantaged rural area. The profile of e-cigarette use susceptible individuals is different from that of the general population and, thus, may require different preventive strategies. Susceptibility to e-cigarette use was prevalent in the study population. Our analysis indicated that socio-demographic factors (school grade, maternal educational level, money available per month), substance use (tobacco smoking, alcohol consumption), the youth’s perception of e-cigarettes (its attractiveness and harmfulness), and smoking/e-cigarette use status of parents and friends were associated with e-cigarette susceptibility.

Despite the law amendment regulating e-cigarette use in Poland (age limit, ban on advertising, restriction of the use of e-cigarettes in public places), the percentage of e-cigarette users is even higher than that noted previously in a similar population (a study conducted in Piotrkowski district in 2014–2015 vs. the current study: 22% vs. 25.7% of the sample reported ever using e-cigarettes and 27% vs. 31.7% of the respondents indicated e-cigarette use in the past month) [13,32]. In a recently published analysis that was performed among university students from five European countries (Belarus, Lithuania, Poland, Russia, and Slovakia) indicated that 44% of the study population had ever used e-cigarettes (45% in Poland) [33].

In our study, the assessment of e-cigarettes use susceptibility was performed for the young people who have never used these products and among those who declared that they had ever smoked e-cigarettes but did not use them in 30 days preceding the study. This is important from the point of view of promotional activities, which, in order to be effective, should take into account characteristics of each of these groups. The study indicated extremely high percentages of the youth (68% of the never and 78% of the ever e-cigarette users) that were classified as susceptible to e-cigarette use. A significantly lower percentage of people susceptible to the use of e-cigarettes was recorded among the high school students who had not yet tried these products (38%). The results noted in the current study are higher than those observed for young people in the analyses of Bold et al. (2016) and Kwon et al. (2018), wherein 28% and 24% of their respondents, respectively, were classified as susceptible to the use of e-cigarettes [22,23]. Apart from the country-specific factors, definition of the population covered by each study may be a cause of the observed differences. In the case of the study carried out in Piotrkowski district, the population was limited to never and ever e-cigarettes users (included in separate and combined analyses), and could include the respondents who smoked traditional cigarettes (in the past, occasionally, or on a daily basis). The remaining studies included young people who never smoked traditional cigarettes and never used e-cigarettes. If we restricted our population to this criterion, percentage of the susceptible youth (51%) would decrease but it would still be higher when compared to the other studies. In addition, one of the questions used to assess susceptibility was slightly different. In our study, the following question was asked “At any time during the next 12 months, do you think you will use a cigarette?”, whereas in the study by Kwon et al. (2018) their participants had to answer a question “Do you think that you will try an e-cigarettes soon?”, and in the analysis of Bold and et al. (2016) “Do you think you can experiment with e-cigarettes in the future?” [22,23,24,25,26,27,28,29,30,31,32,33,34].

It should also be emphasized that the percentage of young people susceptible to e-cigarette use has been found to be much higher in comparison to the percentage of those susceptible to smoking traditional cigarettes (68% vs. 22% for never users and 78% vs. 57% for ever users) [30]. Both analyses were conducted at the same schools with similar procedures and questions asked (the former study performed between 2014 and 2015 and the current study performed between 2017 and 2018). This can be explained by a different perception of harmfulness, popularity of those products, and social norms related to their use.

The profiles of susceptible to e-cigarette use young people were similar in the analyses performed for the never-users and for those who only experimented with these products. The following main risk factors for susceptibility to the use of e-cigarettes were identified: smoking cigarettes by friends or parents, alcohol consumption, smoking status, higher maternal level of education, and the perception of girls using e-cigarettes as more attractive. In addition, the opinion about harmfulness of e-cigarettes in relation to traditional cigarettes was important in the never-using group. Among the ever users, those in secondary schools were more likely to start using e-cigarettes, whereas those indicating more money per month were less likely to do so. Similarly, in the studies cited above, risky behaviors, including alcohol consumption, and in the study of Kwon et al. (2018), exposure to environmental tobacco smoke at home, increased the susceptibility to the use of e-cigarettes [23]. Some predictors of e-cigarette susceptibility are similar to those identified for traditional cigarette smoking (having smoking friends or perceiving smoking girls as more attractive) [30]. However, some differences have also been noted (attending secondary school and a higher maternal level of education as a predictor of susceptibility to e-cigarette use and an older age, as well as a lower maternal educational level as predictors of susceptibility to smoking) [30]. These differences suggest that the youth who are attracted to e-cigarettes might not be identical to those who are attracted to traditional cigarettes. This indicates that prevention strategies should be tailored depending on the nicotine product that is addressed.

The current study analyzed comprehensively the prevalence and correlates of e-cigarette use susceptibility among young people. It is worth noting that this is the first time such an analysis has been performed in Poland. All the gathered data are extremely important from the public health point of view, and provide potential ways to change the behavior of young people with regard to the use of e-cigarettes. It is also worth emphasizing that the study was conducted in a rural population, characterized by a much lower development and health index than those for the general population. Rural populations, compared to those living in large cities, are less frequently covered by comprehensive scientific research, as well as educational or intervention measures. The advantages of the study also include the standardized questionnaire and the classification of variables similar to those in other studies (which allows for the comparison and changes over the time). A high response rate (over 85%) observed in the current study also needs to be pointed out.

Nevertheless, the study is also the subject of some limitations. Firstly, due to the cross-sectional nature of the study, causal inference on the relationship between susceptibility to e-cigarette use and various factors is not warranted. Secondly, the analysis covered the adolescents attending schools in Piotrkowski district (which, as mentioned previously, is recognized as a socio-economically disadvantaged rural area). Thus, the observed associations can be specific for the analyzed population but different from the ones noted for the general population. Nonetheless, the observed predictors were similar to those noted in other studies in the same field [22,23,24,27]. Furthermore, the self-reported measures (cigarette smoking and the use of e-cigarettes) could be a subject of bias. In addition, despite the fact that many variables were considered in the analyses, some important factors may have been omitted. These include psychosocial conditions, parental support, curiosity, and school achievements, which were not the subject of the undertaken analysis, and which, in the light of existing research, may affect susceptibility to e-cigarette use.

## 5. Conclusions

The current study indicates that susceptibility to e-cigarette use is highly prevalent among the secondary and high school students in a socio-economically disadvantaged rural area in Poland. Personal, social, and environmental factors identified as predictors of e-cigarette susceptibility should be included in prevention programs for the youth to make them effective. It is crucial to incorporate such programs as early as possible as a part of school curriculum. Moreover, they should cover the broad spectrum of risky behaviors and aim at changing social norms around e-cigarette use (to minimize attractiveness of these products). The youth should be equipped with knowledge on health risks related to e-cigarette use and skills to resist their use. Finally, there is a need to increase awareness of the law and to enforce the existing legislation.

Future studies should focus on a variety of populations, as e-cigarette use susceptibility can be country- or even region-specific, depending on characteristics of the population itself, social norms, legislation, and preventive measures. They should also consider a broad spectrum of factors including psychosocial conditions, curiosity, and peer and parental relationships. Furthermore, the effectiveness of the legislation and educational as well as interventional activities should be evaluated in the context of prevention from e-cigarette initiation and experimentation.

## Figures and Tables

**Table 1:** Factors associated with susceptibility to e-cigarette use among the secondary and high school students from Piotrkowski district—an analysis for the never e-cigarette users.

Characteristic	Characteristics of Never E-Cigarette Users	Proportion of Those Susceptible to E-Cigarette Use	Crude		Adjusted	
	*n* (%)	*n* (%)	OR	95% CI	OR	95% CI
All	1054 (100)	713 (67.6)				
Gender						
Male	596 (56.6)	397 (66.6)	0.88	0.68–1.14		
Female	458 (43.4)	316 (69.0)	Ref.			
Age (in years) #						
≤15	743 (70.8)	594 (79.9)	5.78 ***	4.13–8.10		
16–17	111 (10.5)	37 (33.3)	0.73	0.45–1.18		
≥18	196 (18.7)	80 (40.8)	Ref.			
School grade						
Secondary	743 (70.5)	594 (79.9)	6.43 ***	4.81–8.60	1.12	0.55–4.00
High	311 (29.5)	119 (38.3)	Ref.		Ref.	
Mother’s education						
Low	469 (45.0)	291 (62.0)	Ref.		Ref.	
Medium	310 (29.8)	202 (65.2)	1.13	0.84–1.52	2.72 **	1.48–5.02
High	263 (25.2)	214 (81.4)	2.70 ***	1.89–3.89	2.71 *	1.15–6.39
Father’s education						
Low	530 (50.9)	367 (69.2)	Ref.		Ref.	
Medium	283 (27.1)	148 (52.3)	0.49 ***	0.36–0.66	0.67	0.34–1.07
High	229 (22.0)	194 (84.7)	2.47 ***	1.65–3.71	1.97	0.76–5.09
Money available per month						
<100 PLN	610 (58.8)	425 (69.7)	Ref.			
≥100 PLN	427 (41.2)	279 (65.3)	0.83	0.64–1.08		
Parental smoking						
None	538 (51.3)	342 (63.6)	Ref.		Ref.	
One or both	511 (48.7)	368 (72.0)	1.51 **	1.16–1.95	3.03 ***	1.61–5.69
Parental e-cigarettes use						
None	701 (67.3)	460 (65.6)	Ref.		Ref.	
One or both	340 (32.7)	247 (72.6)	1.40 *	1.05–1.86	1.09	0.52–1.40
Friends’ smoking						
None	244 (23.2)	134 (54.9)	Ref.		Ref.	
Some	810 (76.8)	579 (71.5)	2.05 ***	1.53–2.76	1.98 *	1.06–3.70
Friends’ e-cigarette use						
None	88 (11.7)	53 (60.2)	Ref.		Ref.	
Some	667 (88.3)	474 (71.1)	1.62 *	1.02–2.56	1.60	0.75–3.45
Ban on smoking at home						
Yes	443 (42.1)	281 (63.4)	Ref.		Ref.	
No	609 (57.9)	432 (70.9)	1.40 **	1.08–1.81	1.03	0.44–1.36
Ban on smoking at school						
Yes	671 (64.0)	459 (68.4)	Ref.			
No	377 (36.0)	252 (66.8)	0.94	0.73–1.24		
Ban on e-cigarette use at school						
Yes	444 (42.3)	268 (60.4)	Ref.		Ref.	
No	607 (57.7)	445 (73.3)	1.82 ***	1.40–2.36	1.09	0.69–1.72
Alcohol consumption						
Non-drinker	343 (32.6)	171 (49.9)	Ref.		Ref.	
Moderate	251 (23.9)	180 (71.7)	2.56 ***	1.81–3.62	2.94 ***	1.50–5.76
Binge	458 (43.5)	362 (79.0)	3.70 ***	2.72–5.04	2.19 **	1.25–3.82
Tobacco smoking						
Never smoker	461 (43.7)	234 (50.8)	Ref.		Ref.	
Former smoker	312 (29.6)	216 (69.2)	2.14 ***	1.58–2.89	1.56	0.93–2.60
Current smoker	281 (26.7)	263 (93.6)	14.22 ***	8.51–23.75	14.05 ***	3.30–59.81
Girls who use e-cigarettes are:						
More attractive	389 (36.9)	350 (90.0)	7.52 ***	5.22–10.83	4.08 ***	2.05–8.10
Less attractive or no difference	665 (63.1)	363 (54.6)	Ref.		Ref.	
Boys who use e-cigarettes are:						
More attractive	432 (41.3)	343 (79.4)	2.52 ***	1.92–3.38	1.40	0.80–2.47
Less attractive or no difference	615 (58.7)	370 (60.2)	Ref		Ref.	
Perception that smoking is harmful to health						
Yes	924 (88.0)	614 (66.5)	Ref.		Ref.	
No	126 (12.0)	95 (75.4)	1.55 *	1.01–2.37	1.70	0.62–4.66
Relative harmfulness (compared to traditional cigarettes)						
As harmful	458 (43.9)	288 (62.9)	Ref.		Ref.	
Less harmful	508 (48.6)	382 (75.2)	1.75 ***	1.33–2.31	1.80 ***	1.04–3.10
More harmful	78 (7.5)	42 (53.9)	0.68	0.42–1.11	0.96	0.41–2.25

* *p* < 0.05; ** *p* < 0.01; *** *p* < 0.001. # age was not included in the multivariate model as this variable was highly correlated with the school grade. OR: odds ratio. PLN: Polish currency.

**Table 2:** Factors associated with susceptibility to e-cigarette use among the secondary and high school students from Piotrkowski district—an analysis for the ever e-cigarette users.

Characteristic	Characteristics of Ever E-Cigarette Users	Proportion of Those Susceptible to E-Cigarette Use	Crude		Adjusted	
	*n* (%)	*n* (%)	OR	95% CI	OR	95% CI
All	639 (100)	495 (77.5)				
Gender						
Male	342 (53.5)	269 (78.7)	1.16	0.80–1.68		
Female	297 (46.5)	226 (76.1)	Ref.			
Age (in years) #						
≤15	440 (69.3)	355 (80.7)	1.36.	0.86–2.17		
16–17	65 (10.2)	39 (60.0)	0.49	0.26–0.93*		
≥18	130 (20.5)	98 (75.4)				
Secondary	440 (68.9)	355 (80.7)	1.76 **	1.20–2.59	5.56 ***	3.13–10.00
High	199 (31.1)	140 (70.4)	Ref.		Ref.	0.10–0.32
School grade						
Secondary	440 (68.9)	355 (80.7)	Ref.		Ref.	
High	199 (31.1)	140 (70.4)	0.57 **	0.39–0.84	0.18 ***	0.10–0.32
Mother’s education						
Low	291 (46.9)	199 (68.4)	Ref.		Ref.	
Medium	190 (30.6)	164 (86.3)	2.92 ***	1.80–4.23	5.43 ***	2.85–10.36
High	140 (22.5)	121 (86.4)	2.94 ***	1.71–5.08	4.37 ***	2.08–9.17
Father’s education						
Low	310 (49.7)	243 (78.4)	Ref.			
Medium	213 (34.1)	160 (75.1)	0.83	0.55–1.26		
High	101 (16.2)	82 (81.2)	1.19	0.67–2.10		
Money available per month						
<100 PLN	393 (63.5)	324 (82.4)	Ref.		Ref.	
≥100 PLN	226 (36.5)	158 (69.9)	0.49 **		0.37 ***	0.22–0.61
Parental smoking						
None	306 (48.3)	223 (72.9)	Ref.		Ref.	
One or both	327 (51.7)	268 (82.0)	1.69 **	1.16–2.47	2.22 **	1.33–3.71
Parental e-cigarettes use						
None	418 (67.9)	316 (75.6)	Ref.			
One or both	198 (32.1)	161 (81.3)	1.40	0.92–2.14		
Friends’ smoking						
None	122 (19.1)	76 (62.3)	Ref.		Ref.	
Some	517 (80.9)	419 (81.0)	2.59 ***	1.69–3.97	2.20 **	1.20–4.04
Friends’ e-cigarette use						
None	6 (1.1)	5 (83.3)	Ref.			
Some	521 (98.9)	405 (77.7)	0.69	0.08–6.07		
Ban on smoking at home						
Yes	250 (39.2)	203 (81.2)	Ref.			
No	388 (60.8)	292 (75.3)	0.70	0.48–1.04		
Ban on smoking at school						
Yes	400 (62.6)	311 (77.8)	Ref.			
No	239 (37.4)	184 (77.0)	0.96	0.65–1.40		
Ban on e-cigarette use at school						
Yes	254 (40.0)	198 (78.0)	Ref.			
No	381 (60.0)	293 (76.9)	0.94	0.64–1.38		
Alcohol consumption						
Non-drinker	155 (24.4)	94 (60.6)	Ref.		Ref.	
Moderate	158 (24.8)	141 (89.2)	5.38 ***	2.69–9.80	4.24 ***	2.03–8.84
Binge	323 (50.8)	258 (79.9)	2.57 ***	1.69–3.93	1.54	0.90–2.61
Tobacco smoking						
Never smoker	221 (34.6)	142 (64.3)	Ref.		Ref.	
Former smoker	237 (37.1)	184 (77.6)	1.93 **	1.28–2.92	2.67 ***	1.56–4.56
Current smoker	181 (28.3)	169 (93.4)	7.84 ***	4.09–15.00	11.32 ***	6.04–21.13
Girls who use e-cigarettes are:						
More attractive	205 (32.1)	187 (91.2)	4.25 ***	2.51–7.21	2.88 **	1.42–5.86
Less attractive or no difference	434 (67.9)	308 (71.0)	Ref.		Ref.	
Boys who use e-cigarettes are:						
More attractive	231 (36.3)	183 (79.2)	1.18	0.80–1.75		
Less attractive or no difference	406 (63.7)	310 (76.4)	Ref			
Perception that smoking is harmful to health						
Yes	557 (87.2)	435 (78.1)	Ref.			
No	82 (12.8)	60 (73.2)	0.76	0.45–1.30		
Relative harmfulness (compared to traditional cigarettes)						
As harmful	254 (40.0)	198 (78.0)	Ref.			
Less harmful	318 (50.1)	246 (77.4)	0.97	0.65–1.44		
More harmful	63 (9.9)	48 (76.2)	0.91	0.47–1.74		

* *p* < 0.05; ** *p* < 0.01; *** *p* < 0.001. # age was not included in the multivariate model as this variable was highly correlated with the school grade.

## Supplementary Materials

The following are available online at https://www.mdpi.com/1660-4601/16/20/3935/s1, Table S1: Factors associated with susceptibility to e-cigarette use among the secondary and high school students from Piotrkowski district—analysis for never and ever e-cigarette users.

## References

[1] Glasser A., Abudayyeh H., Cantrell J., Niaura R. (2018). Patterns of E-Cigarette Use Among Youth and Young Adults: Review of the Impact of E-Cigarettes on Cigarette Smoking. Nicotine Tob. Res..

[2] Murthy V.H. (2017). E-Cigarette Use Among Youth and Young Adults: A Major Public Health Concern. JAMA Pediatr..

[3] Perikleous E.P., Steiropoulos P., Paraskakis E., Constantinidis T.C., Nena E. (2018). E-Cigarette Use Among Adolescents: An Overview of the Literature and Future Perspectives. Front. Public Health.

[4] U.S. Department of Health and Human Services (2016). E-Cigarette Use Among Youth and Young Adults. A Report of the Surgeon General.

[5] Kong G., Morean M.E., Cavallo D.A., Camenga D.R., Krishnan-Sarin S. (2015). Reasons for electronic cigarette experimentation and discontinuation among adolescents and young adults. Nicotine Tob. Res..

[6] Grana R.A., Ling P.M. (2014). Smoking revolution: A content analysis of electronic cigarette retail websites. Am. J. Prev. Med..

[7] Christie D., Viner R. (2005). ABC of adolescence: Adolescent development. BMJ.

[8] Montaño D.E., Kasprzyk D., Glanz K., Rimer B.K., Viswanath K. (2015). Theory of reasoned action theory of planned behavior and the integrated behavioral model. Health Behavior: Theory Research and Practice.

[9] Choi K., Fabian L., Mottey N., Corbett A., Forster J. (2012). Young adults’ favorable perceptions of snus. dissolvable tobacco products. and electronic cigarettes: Findings from a focus group study. Am. J. Public Health.

[10] Peters R.J., Meshack A., Lin M.T., Hill M., Abughosh S. (2013). The social norms and beliefs of teenage male electronic cigarette use. J. Ethn. Subst. Abuse.

[11] Ambrose B.K., Rostron B.L., Johnson S.E., Portnoy D.B., Apelberg B.J., Kaufman A.R., Choiniere C.J. (2014). Perceptions of the relative harm of cigarettes and e-cigarettes among U.S. Youth. Am. J. Prev. Med..

[12] Cooper M., Case K.R., Loukas A., Creamer M.R., Perry C.L. (2016). E-cigarette dual users exclusive users and perceptions of tobacco products. Am. J. Health Behav..

[13] Kaleta D., Wojtysiak P., Polańska K. (2016). Use of electronic cigarettes among secondary and high school students from a socially disadvantaged rural area in Poland. BMC Public Health.

[14] Agaku I.T., Ayo-Yusuf O.A. (2014). The effect of exposure to pro-tobacco advertising on experimentation with emerging tobacco products among U.S. adolescents. Health Educ. Behav..

[15] Giovacchini C.X., Pacek L., McClernon F.J., Que L.G. (2017). Use and perceived risk of electronic cigarettes among North Carolina middle and high school students. N. C. Med. J..

[16] Lee Y.O., Hebert C.J., Nonnemaker J.M., Kim A.E. (2015). Youth tobacco product use in the United States. Pediatrics.

[17] Khan T., Baker D.C., Quinn C.M., Huang J., Chaloupka F.J. (2014). Changes in E-Cigarette Availability over Time in the United States: 2010–2012—A BTG Research Brief Chicago, IL, Bridging the Gap Program.

[18] Eadie D., Stead M., MacKintosh A.M., MacDonald L., Purves R., Pearce J., Tisch C., van der Sluijis W., Amos A., MacGregor A. (2015). E-cigarette marketing in UK stores: An observational audit and retailers’ views. BMJ Open.

[19] Morain S.R., Malek J. (2017). Minimum age of sale for tobacco products and electronic cigarettes: Ethical acceptability of US “tobacco 21 laws”. Am. J. Public Health.

[20] Goniewicz M.L., Smith D.M., Edwards K.C., Blount B.C., Caldwell K.L., Feng J., Wang L., Christensen C., Ambrose B., Borek N. (2018). Comparison of Nicotine and Toxicant Exposure in Users of Electronic Cigarettes and Combustible Cigarettes. JAMA Netw. Open.

[21] Pierce J.P., Choi W.S., Gilpin E.A., Farkas A.J., Merritt R.K. (1996). Validation of susceptibility as a predictor of which adolescents take up smoking in the United States. Health Psychol..

[22] Bold K.W., Kong G., Cavallo D.A., Camenga D.R., Krishnan-Sarin S. (2016). E-Cigarette Susceptibility as a Predictor of Youth Initiation of E-Cigarettes. Nicotine Tob. Res..

[23] Kwon E., Seo D.C., Lin H.C., Chen Z. (2018). Predictors of youth e-cigarette use susceptibility in a U.S. nationally representative sample. Addict. Behav..

[24] Barrington-Trimis J.L., Liu F., Unger J.B., Alonzo T., Cruz T.B., Urman R., Pentz M.A., Berhane K., McConnell R. (2019). Evaluating the predictive value of measures of susceptibility to tobacco and alternative tobacco products. Addict. Behav..

[25] Bold K.W., Krishnan-Sarin S., Stoney C.M. (2018). E-cigarette use as a potential cardiovascular disease risk behavior. Am. Psychol..

[26] Lozano P., Arillo-Santillán E., Barrientos-Gutiérrez I., Zavala-Arciniega L., Reynales-Shigematsu L.M., Thrasher J.F. (2019). E-cigarette use and its association with smoking reduction and cessation intentions among Mexican smokers. Salud Publica Mex..

[27] Nicksic N.E., Barnes A.J. (2019). Is susceptibility to E-cigarettes among youth associated with tobacco and other substance use behaviors one year later? Results from the PATH study. Prev. Med..

[28] Goniewicz M.L., Zielinska-Danch W. (2012). Electronic Cigarette Use Among Teenagers and Young Adults in Poland. Pediatrics.

[29] Goniewicz M.L., Gawron M., Nadolska J., Balwicki L., Sobczak A. (2014). Rise in ElectronicCigarette Use among Adolescents in Poland. J. Adolesc. Health.

[30] Polańska K., Wojtysiak P., Bąk-Romaniszyn L., Kaleta D. (2016). Susceptibility to cigarette smoking among secondary and high school students from a socially disadvantaged rural area in Poland. Tob. Induc. Dis..

[31] Kaleta D., Polanska K., Wojtysiak P., Szatko F. (2017). Involuntary Smoking in Adolescents, Their Awareness of Its Harmfulness, and Attitudes towards Smoking in the Presence of Non-Smokers. Int. J. Environ. Res. Public Health.

[32] The Act of 22.07.2016 on the Amendment of the Law on Health Protection from the Consequences of Tobacco and Tobacco Products. http://prawo.sejm.gov.pl/isap.nsf/DocDetails.xsp?id=WDU20160001331.

[33] Brożek G.M., Jankowski M., Lawson J.A., Shpakou A., Poznański M., Zielonka T.M., Klimatckaia L., Loginovich Y., Rachel M., Gereová J. (2019). The Prevalence of Cigarette and E-cigarette Smoking Among Students in Central and Eastern Europe—Results of the YUPESS Study. Int. J. Environ. Res. Public Health.

[34] Strong D.R., Hartman S.J., Nodora J., Messer K., James L., White M., Portnoy D.B., Choiniere C.J., Vullo G.C., Pierce J. (2015). Predictive Validity of the Expanded Susceptibility to Smoke Index. Nicotine Tob. Res..

