# Supplementary material for: Predictors of E-Cigarette Use Susceptibility—A Study of Young People from a Socio-Economically Disadvantaged Rural Area in Poland
Source: Int J Environ Res Public Health. 2019 Oct 16;16(20):3935. doi: 10.3390/ijerph16203935 (PMC6843563; doi:10.3390/ijerph16203935)
Supplement: Supplementary file 1 [file ijerph-16-03935-s001.pdf]

International Journal of
Environmental Research and Public Health

MDPI

Table S1. Factors associated with susceptibility to e-cigarette use among the secondary and high school students from Piotrkowski district—analysis for never and ever e-cigarette users.

Characteristic	Characteristics of Never and Ever E-Cigarette Users *n* = 1693	Proportion of Susceptible to E-Cigarette Use *n* = 1208 (71.4%)	Crude		Adjusted	
	n (%)	*n* (%)	OR	95 % CI	OR	95 % CI
		Gender				
Male	938 (55.4)	666 (71.0)	0.96	0.78–1.19		
Female	755 (44.6)	542 (71.8)	Ref.			
		Age (in years)				
≤15	1183 (70.2)	949 (80.2)	3.37 ***	2.60–4.38		
16–17	176 (10.4)	76 (43.2)	0.63 **	0.43–0.91		
≥18	326 (19.3)	178 (54.6)	ref.			
		School grade				
Secondary	1183 (69.9)	949 (80.2)	3.93 ***	3.14–4.92	6.2 5***	4.17–10.00
High	510 (30.1)	259 (50.8)	Ref.		Ref.	
		Mother's education				
Low	760 (45.7)	490 (64.5)	Ref.		Ref.	
Medium	500 (30.1)	366 (73.2)	1.51 ***	1.17–1.93	3.97 ***	2.55–6.18
High	403 (24.2)	335 (83.1)	2.71 ***	2.01–3.67	3.96 ***	2.16–7.27
		Father's education				
Low	840 (50.4)	610 (72.6)	Ref.		Ref.	
Medium	496 (29.8)	308 (62.1)	0.62 ***	0.49-0.72	0.86	0.57–1.29
High	330 (19.8)	276 (83.6)	1.93 ***	1.39–2.68	1.70	0.87–3.33
		Money available per month				
<100 PLN	1003 (60.6)	749 (74.7)	Ref.		Ref.	
≥100 PLN	653 (39.4)	437 (66.9)	0.69 ***	0.55–0.85	0.47 ***	0.31–0.69
		Parental smoking				
None	844 (50.2)	565 (66.9)	Ref.		Ref.	
One or both	838 (49.8)	636 (75.9)	1.55 ***	1.26–1.93	2.35 ***	1.63–3.38
		Parental e-cigarettes use				
None	1119 (67.5)	776 (69.3)	Ref.		Ref.	
One or both	538 (32.5)	408 (75.8)	1.39 **	1.10–1.75	1.09	0.66–1.42
		Friends' smoking				

None	366 (21.6)	210 (57.4)	Ref.		Ref.	
Some	1327 (78.4)	998 (75.2)	2.25 ***	1.77–2.87	1.88 **	1.22–2.89
Friends’ e-cigarette use						
None	94 (7.3)	58 (61.7)	Ref.		Ref.	
Some	1188 (92.7)	879 (74.0)	1.77 **	1.14–2.73	1.33	0.70–2.52
Ban on smoking at home						
Yes	693 (41.0)	484 (69.8)	Ref.			
No	997 (59.0)	724 (72.6)	1.15	0.92-1.42		
Ban on smoking at school						
Yes	1071 (63.5)	770 (71.9)	Ref.			
No	616 (36.5)	436 (70.8)	0.95	0.76–1.18		
Ban on e-cigarettes use at school						
Yes	698 (41.4)	466 (66.8)	Ref.		Ref.	
No	988 (58.6)	738 (74.7)	1.47 ***	1.19–1.82	1.04	0.68–1.46
Alcohol consumption						
Non-drinker	498 (29.5)	265 (53.2)	Ref.		Ref.	
Moderate	409 (24.2)	321 (78.5)	3.21 ***	2.39–4.31	3.29 ***	2.07–5.25
Binge	781 (46.3)	620 (79.4)	3.39 ***	2.64–4.34	1.88 **	1.27–2.80
Tobacco-smoking						
Never smokers	682 (40.3)	376 (55.1)	Ref.		Ref.	
Former smokers	549 (32.4)	400 (72.9)	2.18 ***	1.72–2.78	2.17 ***	1.49–3.15
Current smokers	462 (27.3)	432 (93.5)	11.72 ***	7.85–17.50	13.17 ***	9.79–24.56
Girls who use e–cigarettes are:						
More attractive	594 (35.1)	537 (90.4)	6.01 ***	4.45–8.11	3.23 ***	2.03–5.15
Less attractive or no difference	1099 (64.9)	671 (61.1)	Ref.		Ref.	
Boys who use e–cigarettes are:						
More attractive	663 (39.4)	526 (79.3)	1.93 ***	1.53–2.42	1.38	0.94–2.04
Less attractive or no difference	1021 (60.6)	680 (66.6)	Ref		Ref.	
Perception that smoking is harmful to health						
Yes	1481 (87.7)	1049 (70.8)	Ref.			
No	208 (12.3)	155 (74.5)	1.20	0.86–1.68		
Relative harmfulness (comparing to traditional cigarettes)						
As harmful	712 (42.4)	486 (68.3)	Ref.		Ref.	
Less harmful	826 (49.2)	628 (76.0)	1.47 ***	1.18–1.85	1.50 *	1.05–2.15
More harmful	141 (8.4)	90 (63.8)	0.82	0.56–1.20	0.89	0.64–2.21

			Use of e–cigarettes			
Never	1054 (62.3)	713 (67.6)	Ref.		Ref.	
Ever	639 (37.7)	495 (77.5)	1.64 ***	1.31–2.06	1.30	0.91–1.84

* $p < 0.05$; ** $p < 0.01$; *** $p < 0.001$. # age was not included in multivariate model as this variable was highly correlated with school grade.
